# Supplementary material for: DMRforPairs: identifying Differentially Methylated Regions between unique samples using array based methylation profiles
Source: BMC Bioinformatics. 2014 May 15;15:141. doi: 10.1186/1471-2105-15-141 (PMC4046028; doi:10.1186/1471-2105-15-141)
Supplement: Additional file 2 — DMRforPairs output for the comparison of A431-MCF7 and NA17018-NA17105. Please start from the HTML files in each folder. Available via the BMC Bioinformatics website. [file 1471-2105-15-141-S2.zip › 1394847754114233_MOESM2_ESM/A431_MCF7/figures/22822.pdf]

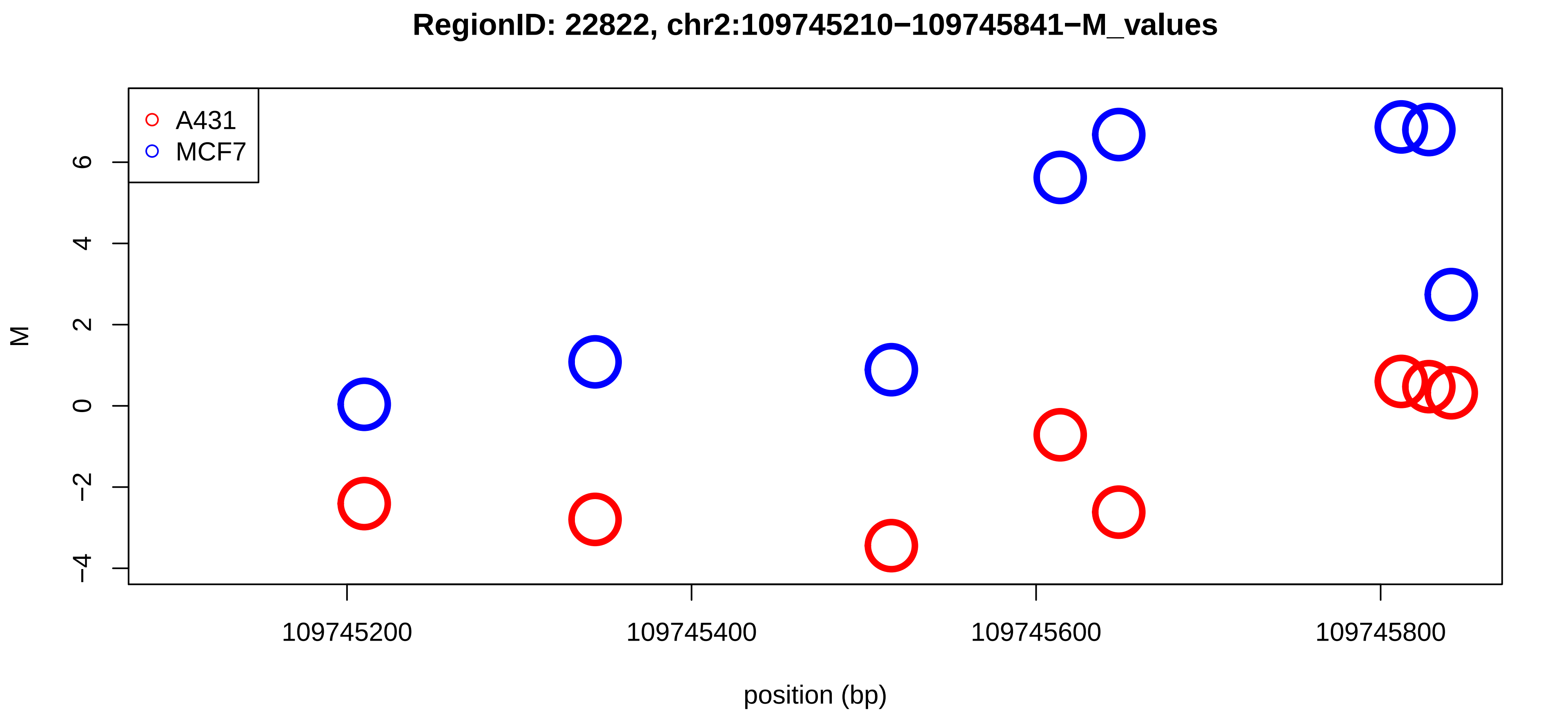
RegionID: 22822, chr2:109745210−109745841−M_values
A431
MCF7
M
−4
−2
0
2
4
6
109745200
109745400
109745600
109745800
position (bp)

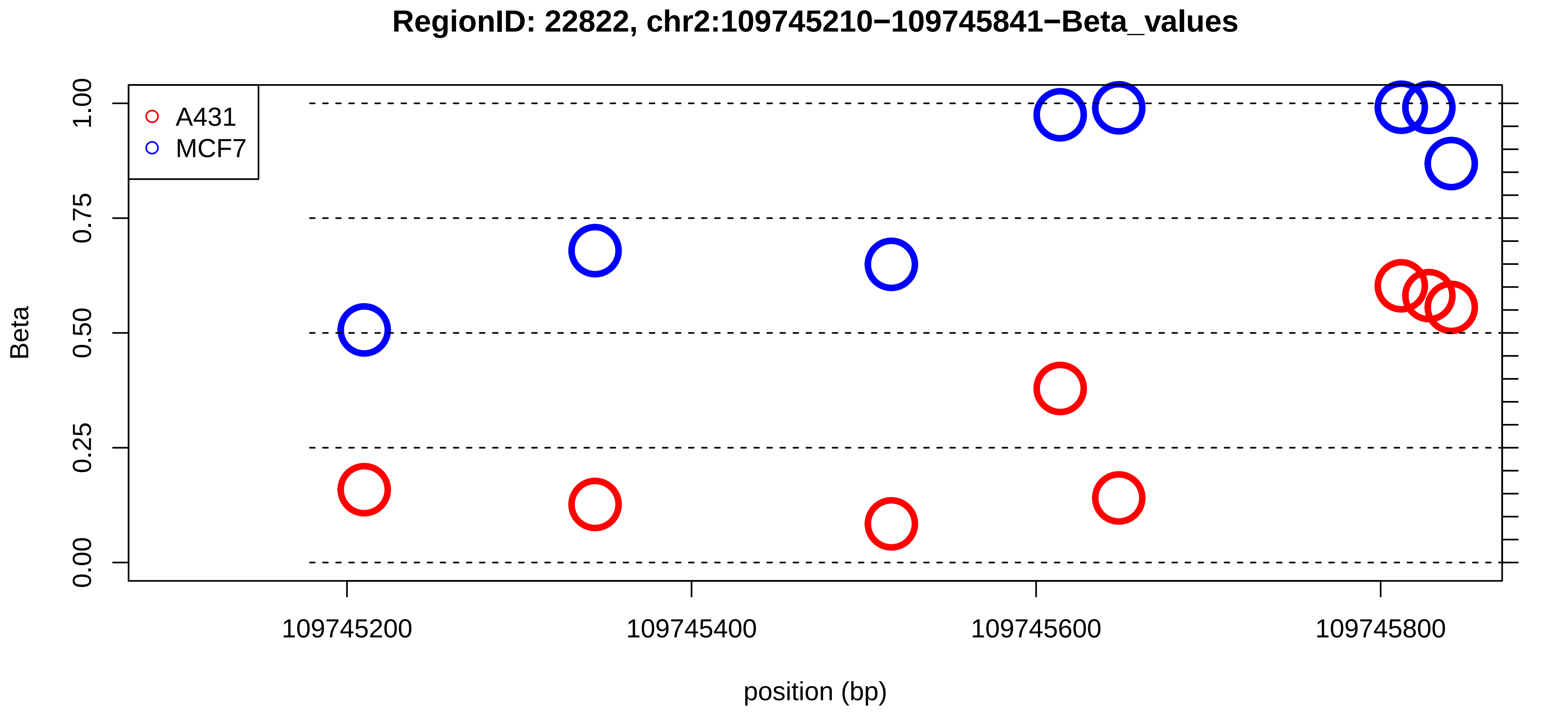
RegionID: 22822, chr2:109745210−109745841−Beta_values
A431
MCF7
Beta
0.00
0.25
0.50
0.75
1.00
109745200
109745400
109745600
109745800
position (bp)

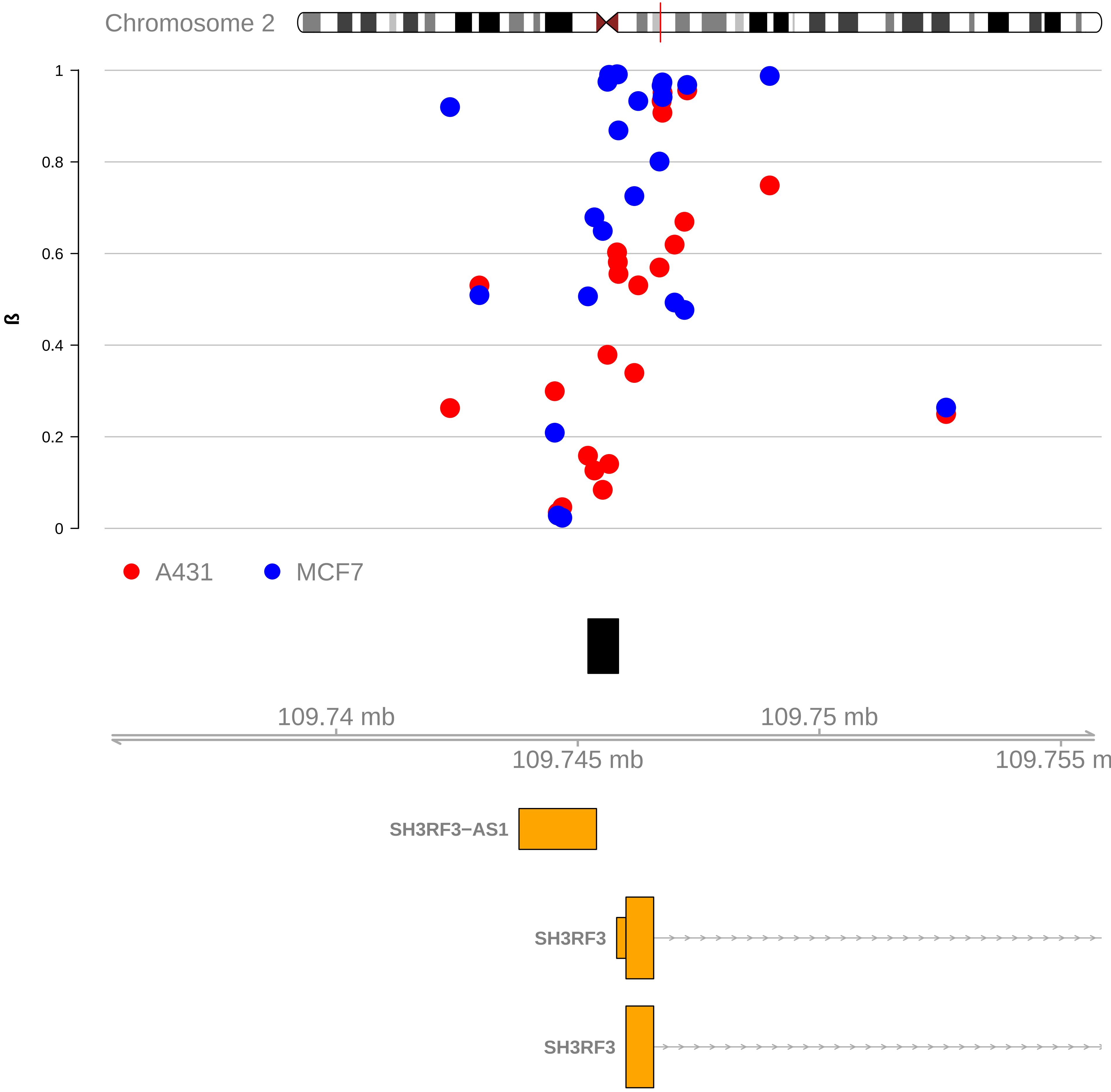
Chromosome 2
ß
1
0.8
0.6
0.4
0.2
0
A431
MCF7
109.74 mb
109.745 mb
109.75 mb
109.755 m
SH3RF3−AS1
SH3RF3
SH3RF3
